# Type 1 diabetes, ageing and frailty: an underexplored intersection

**DOI:** 10.1007/s00125-026-06681-x

**Published:** 2026-02-17

**Authors:** Giuseppe Maltese, Janaka Karalliedde, Jugdeep Dhesi, Srikanth Bellary

**Affiliations:** 1 Department of Diabetes and Endocrinology, Epsom and St Helier University Hospitals NHS Trust, London, UK; 2 School of Cardiovascular Medicine & Metabolic Sciences, King’s College London, London, UK; 3 Department of Ageing and Health, Guy’s and St Thomas NHS Foundation Trust, London, UK; 4 School of Life Course and Population Sciences, Faculty of Life Sciences and Medicine, King’s College London, London, UK; 5 College of Health and Life Sciences, Aston University, Birmingham, UK; 6 Department of Diabetes and Endocrinology, University Hospitals Birmingham NHS Foundation Trust, Birmingham, UK

**Keywords:** Ageing, Cognitive impairment, Diabetes technology, Frailty, Geriatrics, Hypoglycaemia, Multimorbidity, Older people, Review, Sarcopenia, Type 1 diabetes

## Abstract

Over recent decades, the life expectancy of individuals with type 1 diabetes has steadily improved due to advances in therapies that enhance metabolic control alongside better prevention and management of complications. However, this extended survival brings new challenges. Type 1 diabetes, through sustained hyperglycaemia and recurrent hypoglycaemia, may act as an accelerator of ageing, predisposing individuals to the development of geriatric syndromes such as frailty. Frailty, defined as a state of reduced physiological reserve that heightens susceptibility to stressors and impairs the ability to restore homeostasis after acute events, has emerged as a recognised complication of diabetes and has been associated with several adverse outcomes including increased risks of hypoglycaemia, hospitalisation, disability, institutionalisation and death. The putative pathophysiology of frailty in type 1 diabetes is complex and multifactorial. It reflects the direct effects of chronic exposure to hyperglycaemia and consequent micro- and macrovascular complications, superimposed on age- and diabetes-related hormonal changes. Additional contributors include sarcopenia, cognitive decline and other comorbidities. Currently, most of the literature on diabetes and frailty focuses on type 2 diabetes, while the relationship with type 1 diabetes and the impact on outcomes remain to be fully elucidated. In this review we discuss the growing evidence on the link between frailty and type 1 diabetes, explore its underlying pathophysiological mechanisms, discuss assessment and treatment strategies, and highlight the key knowledge gaps and suggest future research directions in this evolving field.

**Graphical Abstract:**

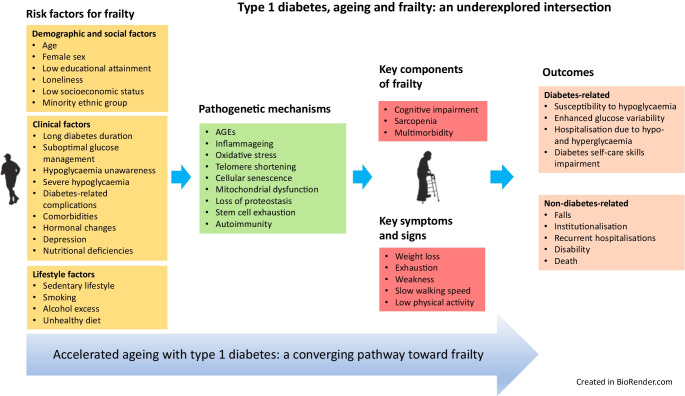

**Supplementary Information:**

The online version contains slideset of the figures for download  available at 10.1007/s00125-026-06681-x.

## Introduction

The number of older adults living with type 1 diabetes is growing, largely driven by therapeutic advancements in diabetes care and improved life expectancy [1]. According to a recent population-based study, over a period of nearly 30 years, the global prevalence of type 1 diabetes among adults aged 65 years and older has increased by 28.5%, while the mortality rate has declined by 25.3%, reflecting significant improvements in survival [2]. However, the achievement of longevity may come with an increased risk of age-related comorbidities, functional and cognitive impairments, frailty and disability [3].

Although the association between frailty and diabetes is well recognised, the body of literature examining this relationship rarely distinguishes between type 1 diabetes and type 2 diabetes [4]. In most studies in this area, the type of diabetes is not reported [4]. This lack of distinction is an important limitation as, despite some common characteristics, the pathophysiological mechanisms underlying frailty in type 1 diabetes may be different from those in type 2 diabetes and remain unexplored. Similarly, the impact of frailty on glucose levels, natural history of diabetes-related complications and other health outcomes in individuals with type 1 diabetes is yet to be investigated.

In this review, we explore the relationship between type 1 diabetes and frailty, examine the underlying mechanisms, discuss frailty assessment tools and highlight strategies to prevent its onset and progression and favour regression. Finally, we suggest future clinical and research directions. Although the primary focus of our review is on older individuals, the concepts we describe may also be applicable to younger people. In fact, frailty can also occur at a young age, particularly when type 1 diabetes is associated with complications and comorbidities that contribute to accelerated ageing.

## Definition of frailty

Frailty is defined as a state of increased vulnerability to stressors due to a reduction in physiological reserve resulting from decline across multiple organ systems [5–7]. It is associated with unfavourable health outcomes, including falls, hospitalisation, disability, dependence and mortality, and represents a concerning public health problem worldwide [8]. Importantly, frailty is a dynamic and potentially reversible condition, characterised by bidirectional transitions between states of fitness and varying degrees of severity (mild, moderate and severe) [9].

Frailty is mainly conceptualised according to two established models: as a syndrome or as an accumulation of health deficits [10]. Both models have been validated in epidemiological studies and proven to be associated with a broad range of adverse outcomes [11].

The Fried frailty phenotype describes frailty as a clinical syndrome characterised by five key features: exhaustion (self-reported based on two items from the Center for Epidemiological Studies Depression Scale), weakness (weak grip strength), slow gait speed (lowest quintile of gait speed stratified by sex and height), low physical activity levels (low energy expenditure, based on physical activity questionnaire) and unintentional weight loss (self-reported weight loss of ≥5% in past year). Based on the number of features present, individuals are identified as robust (none), pre-frail (one or two) or frail (three or more). According to two large cohort milestone studies, the Longitudinal Aging Study Amsterdam, with 15 years of follow-up and the Invecchiare in Chianti (InCHIANTI) study, with 9 years of follow-up, the first physical feature of frailty to occur is exhaustion, followed by slowness, low physical activity levels and weakness [12].

Alternatively, the cumulative deficit model defines frailty as a state of health decline due to the accumulation of age-related deficits [7]. These deficits include long-term conditions, cognitive and physical impairments, and clinical signs, symptoms and abnormal laboratory investigations obtained through clinical assessments or from medical records. The frailty index, calculated as the proportion of accumulated deficits from a total number of health variables, can range from 0 to 1, with higher scores indicating more severe frailty. A minimum of 30–40 deficits are required for an index to be valid [13]. A frailty index >0.70 is associated with a high mortality risk [13].

Frailty is highly prevalent among individuals with diabetes. A systematic review that included 118 studies and over 1.3 million participants reported that the prevalence of frailty in community-dwelling individuals with diabetes ranged from approximately 10% to 25%, with a median prevalence of 13% (IQR 9–21%), when measured using the frailty phenotype [14]. However, estimates varied widely depending on the frailty measure used and study population [14]. Notably, 79% of studies did not specify the type of diabetes, limiting the ability to make conclusions specific to type 1 or type 2 diabetes [14]. The prevalence of frailty in younger individuals with type 1 diabetes is unknown.

## Relationship between diabetes and frailty

The relationship between type 2 diabetes and frailty is known to be bidirectional [15, 16]: diabetes increases the risk of developing frailty but frailty itself may also exacerbate metabolic disturbances and insulin resistance, contributing to hyperglycaemia [17]. In contrast, the relationship between type 1 diabetes and frailty is assumed to be primarily unidirectional, with long-standing type 1 diabetes, particularly when suboptimally managed, contributing to the development of frailty through mechanisms such as chronic hyperglycaemia, glucose variability, severe hypoglycaemia and complications. At present, there is no evidence to suggest that frailty may trigger the development of type 1 diabetes in older adults.

As individuals with type 1 diabetes age, some develop features of the metabolic syndrome, including central adiposity, insulin resistance, hypertension and dyslipidaemia, leading to what is often termed ‘double diabetes’ [18]. Numerous studies have demonstrated that the prevalence of these metabolic traits increases across adulthood and is highest in older age groups [18]. The development of insulin resistance introduces, even in type 1 diabetes, elements of the bidirectional relationship observed in type 2 diabetes and may amplify frailty-related pathways through chronic inflammation and reduced physiological reserve.

Both hyperglycaemia and hypoglycaemia contribute to the development of frailty. Observational and longitudinal studies suggest that in type 2 diabetes persistent hyperglycaemia is associated with greater frailty and increased mortality risk [19, 20]. Similarly, hypoglycaemia has been linked to a significantly elevated risk of frailty, with one study reporting a 44% increased risk [21].

Notably, a U-shaped association between glycaemic management and frailty has been described, with the lowest risk observed with an HbA_1c_ ~60 mmol/mol (7.6%), suggesting that both extremes of glucose levels may be detrimental [22]. Currently, there is no consensus regarding optimal glucose targets for frailty prevention and further studies are required [22].

To date, no studies have investigated the relationship between glucose variability, defined as glucose oscillations over time, and frailty in people with diabetes, particularly type 1 diabetes. Older adults with long-standing type 1 diabetes are more prone to glucose variability than those with type 2 diabetes [23, 24]. Importantly, glucose variability has been shown to correlate to a greater extent than HbA_1c_ with the risk of hypoglycaemia, arrhythmias and increased mortality [25, 26].

## Putative pathophysiological mechanisms linking type 1 diabetes with frailty

**Fig. 1:**
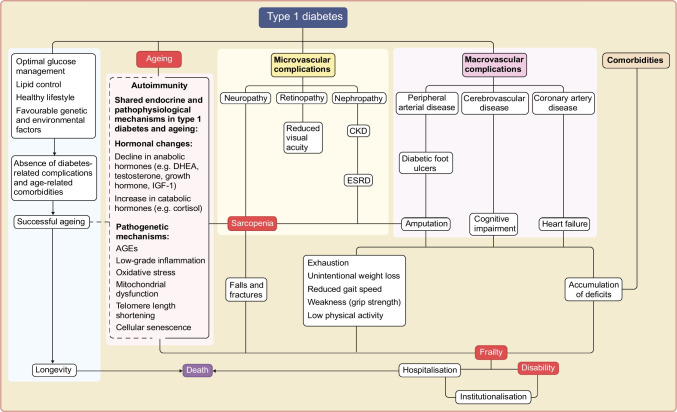
Pathophysiological pathways linking type 1 diabetes, ageing and adverse outcomes. This figure illustrates the interconnected biological and clinical pathways through which type 1 diabetes and ageing contribute to frailty and adverse outcomes. Solid lines represent established biological pathways, whereas dashed lines indicate pathways that can be potentially attenuated but not abolished by optimal metabolic management, healthy lifestyle behaviours and supportive care. Shared endocrine and pathogenic mechanisms include age-related hormonal changes, chronic low-grade inflammation, oxidative stress, mitochondrial dysfunction and cellular senescence. These processes drive the development of microvascular and macrovascular complications, sarcopenia and multiple comorbidities including cardiovascular disease, chronic kidney disease, osteoporosis with increased fracture risk and cognitive impairment, which collectively increase vulnerability to frailty, disability, hospitalisation and mortality. CKD, chronic kidney disease; DHEA, dehydroepiandrosterone; ESRD, end-stage renal disease. This figure is available as part of a downloadable slideset

Frailty is not synonymous with ageing and is not one of its inevitable consequences. Although it is more commonly associated with older age, frailty can occur at any stage of life, particularly in the presence of factors accelerating the ageing process, such as diabetes and its complications, hypertension, dyslipidaemia and other risk factors [27] (Fig. 1). Hormonal changes associated with type 1 diabetes and ageing (discussed in detail later in this review), are also thought to be important contributors (Fig. 1).

Although much of our understanding of the relationship between diabetes and frailty is derived from studies conducted in type 2 diabetes, the underlying mechanisms may potentially be relevant in type 1 diabetes as well.

Diabetes-related microvascular complications are linked to metabolic disturbances, endothelial dysfunction, low-grade inflammation and oxidative stress, all of which lead to vascular damage [28]. These mechanisms are also key drivers of frailty, suggesting a shared pathophysiology possibly mediated by mitochondrial dysfunction [5].

In addition, diabetes-related microvascular complications can compromise physical function, increasing predisposition to frailty. For example, diabetic retinopathy can affect vision, resulting in impaired self-care skills and an increased risk of hypomobility and falls. Diabetic neuropathy can be associated with decreased sensation and gait disturbance, further increasing the risk of falls, fractures, institutionalisation and hospitalisation [29, 30]. Similarly, chronic kidney disease can contribute to frailty, through accelerated muscle mass loss and decreasing functional capacity [31, 32].

Findings from the DIALCAT cohort in Catalonia support the association between microvascular disease and frailty, demonstrating that the presence of microvascular complications in older adults with type 2 diabetes is independently associated with reduced gait speed, even after adjusting for age, sex and cognitive function [33]. Gait speed is a well-established surrogate marker of functional decline and frailty, suggesting that microvascular injury may represent a key mechanistic pathway leading to frailty in diabetes [33]. Although this study was conducted in a cohort of adults with type 2 diabetes, the implications are relevant to individuals with type 1 diabetes who often live with a longer duration of the condition and therefore have a higher risk of microvascular complications.

Beyond vascular complications, type 1 diabetes can predispose to frailty through its impact on skeletal muscle and cognition, two domains addressed in dedicated sections below.

Growing evidence suggests that autoimmunity may play a role in the pathogenesis of frailty, a fascinating hallmark particularly relevant in type 1 diabetes. A Mendelian randomisation study demonstrated that several autoimmune conditions, including type 1 diabetes, rheumatoid arthritis, hypothyroidism and hyperthyroidism, are positively associated with an increased risk of frailty [34]. In this context, other autoimmune pathologies such as coeliac disease and Addison’s disease, frequently seen in people with type 1 diabetes, may further contribute to fatigue, malnutrition and reduced physiological reserves, particularly when undiagnosed or suboptimally managed. The autoimmune component may represent an important differentiating factor in the relationship between frailty and type 1 diabetes compared with type 2 diabetes.

Beyond these diabetes-related factors, ageing per se introduces additional issues, such as low-grade chronic inflammation, mitochondrial dysfunction, oxidative stress and telomere attrition, and can be accompanied by hormonal changes and comorbidities such as cardiovascular disease and osteoporosis [35] (Fig. 1). The state of chronic low-grade inflammation associated with ageing is named ‘inflammageing’ and is characterised by elevated circulating proinflammatory cytokines such as IL-6, TNF-α and C-reactive protein [36]. Inflammageing can influence the progression of diabetes-related complications [37–39] and also mediate sarcopenia and frailty [40–42] (Fig. 1).

## Ageing and type 1 diabetes-related hormonal changes contributing to frailty

Hormonal changes associated with both ageing and type 1 diabetes may contribute to the development and progression of frailty [43]. Age-related declines in anabolic hormones such as testosterone, IGF-1 and oestrogens are known to negatively impact muscle mass, bone density and physical function [44–46]. In individuals with type 1 diabetes, these changes can be amplified by chronic hyperglycaemia, insulin deficiency and diabetes-related complications.

Testosterone concentrations fall by about 1.6% per year after the age of 40 years [47] and longitudinal studies have reported an association with frailty [43, 48]. There is evidence that men with type 1 diabetes have lower testosterone levels than men without diabetes, and low testosterone levels have been linked to reduced muscle strength and increased fat mass [49].

Similarly, circulating growth hormone levels decrease at a rate of 1% per decade from the age of 30 years [50]. Growth hormone exerts many of its anabolic and metabolic effects through IGF-1. IGF-1 regulates the synthesis of transcription factors influencing the expression of genes associated with inflammatory response and cellular autophagy, key mechanisms in frailty [43], and plays a crucial role in skeletal muscle growth, differentiation and regeneration [51]. Age-related changes in the function of IGF-1 are implicated in neuronal senescence and sarcopenia [43]. In individuals with type 1 diabetes, IGF-1 levels are lower than in individuals without diabetes and are independently associated with the risk of sarcopenia [52].

In women, early menopause, which is more common in those with type 1 diabetes, results in an earlier and more pronounced decline in oestrogen levels, thereby increasing the risk of bone fragility [53–55]. Other factors such as vitamin D and the renin–angiotensin system (RAS) also play significant roles in the development and progression of frailty in ageing individuals, particularly those with type 1 diabetes [43]. Vitamin D is essential for calcium metabolism, bone health and muscle function and its deficiency is associated with weakness, bone fragility and increased risk of falls and fractures [40].

In type 1 diabetes, dysregulation of the renin–angiotensin–aldosterone system (RAAS) promotes inflammation, oxidative stress and endothelial dysfunction, leading to micro- and macrovascular disease and arterial stiffness [56–58]. These effects, which are exaggerated with ageing, can potentially contribute to frailty.

## Sarcopenia in type 1 diabetes

Epidemiological studies have shown that from the age of 50 years skeletal mass declines by approximately 1–2% per year, while muscle strength decreases at a rate of about 1.5–3.0% per year [59]. This progressive reduction in muscle mass and function, known as sarcopenia, is associated with increased likelihood of physical disability, falls and mortality [60]. The isolated age-related decline in muscle strength is known as dynapenia.

Sarcopenia is characterised by a reduction in the number and size of type II muscle fibres [61]. According to the European Working Group on Sarcopenia in Older People 2 (EWGSOP2) consensus, sarcopenia is defined through a stepwise approach. Low muscle strength is the key diagnostic criterion and should be assessed first using handgrip strength or chair-rise test. Once low strength is identified, the diagnosis is confirmed by demonstrating low muscle quantity or quality through imaging or body composition techniques. Finally, low physical performance (e.g. gait speed or Short Physical Performance Battery tests) indicates severe sarcopenia.

Sarcopenia is driven by two key factors: (1) anabolic resistance, defined as an attenuated responsiveness of the muscle protein synthesis to anabolic stimuli (e.g. resistance exercise, protein intake); and (2) impaired regeneration, due to reduced satellite cell number and activation, which limits recovery from muscle injury or disuse [40]. Factors contributing to anabolic resistance include insulin resistance, age-related decline in growth hormone and testosterone levels, impaired amino acid sensitivity, mitochondrial dysfunction and inflammageing [40]. Inflammageing interferes with muscle anabolism, disrupting signalling pathways involved in protein synthesis and promoting protein breakdown [62]. There is evidence that in type 1 diabetes mitochondrial dysfunction drives muscle decline through increased oxidative stress, cellular senescence and death [63].

The strong relationship between sarcopenia and diabetes has been studied extensively. However, these studies do not distinguish between type 1 and type 2 diabetes [64].

A large systematic review and meta-analysis involving over 54,000 participants confirmed the bidirectional synergistic relationship between diabetes and sarcopenia and found that sarcopenia occurred more often in those with complications related to diabetes than in those without complications (OR 2.45) [65]. This relationship is mediated through long-standing exposure to hyperglycaemia, enhanced accumulation of AGEs, oxidative stress and chronic inflammation [66].

A key mechanistic distinction exists between types of diabetes. While skeletal muscle insulin resistance is a feature of type 2 diabetes and plays a central role in the pathogenesis of sarcopenia, type 1 diabetes is characterised by insulinopenia, which can promote muscle wasting [67]. Interestingly, there is evidence that the skeletal muscle of adolescents and young adults with type 1 diabetes undergoes changes usually observed in healthy older adults [68]. These alterations, including reduced muscle mass and strength and metabolic dysregulation, seem to be aggravated by the presence of microvascular complications, which are associated with enhanced inflammation, oxidative stress and impaired neuromuscular function [68]. In addition, peripheral arterial disease can accelerate the muscle loss by causing claudication, mobility limitations and reduced physical activity.

In contrast, sarcopenia in type 2 diabetes is more closely linked to ageing, adiposity and comorbidities [69, 70].

Several studies have investigated sarcopenia and muscle dysfunction in individuals with type 1 diabetes across age groups (Table 1). These studies consistently show a higher prevalence of sarcopenia and reduced muscle strength in individuals with type 1 diabetes compared with type 2 diabetes and control individuals [68, 71, 72]. In both types of diabetes and particularly in type 1 diabetes, sarcopenia is associated with neuropathy, reduced renal function, suboptimal glycaemic management, increased risk of falls and poorer quality of life [71, 73].

**Table 1:** Studies exploring the relationship between type 1 diabetes and sarcopenia

Study	Objectives	Type of diabetes	Sample size	Age (years)	Main findings
Mori (2021), cross-sectional study [71]	To examine the prevalence and clinical characteristics of sarcopenia and dynapenia in people with and without diabetes	T1D, T2D	1328 (T1D *n*=177, T2D *n*=645, without diabetes *n*=506)	≥65	Sarcopenia: 42.9% (T1D), 20.9% (T2D), 12.2% (no diabetes) Dynapenia: 11.4% (T1D), 13.9% (T2D), 0.5% (no diabetes) Both linked to older age, neuropathy, lower renal function, poorer physical quality of life and higher fall risk
Shimura (2025), single-centre, cross-sectional study [74]	To investigate prevalence and factors associated with reduced skeletal muscle mass in younger adults with T1D	T1D	99	Mean ± SD: 43 ± 11 (range: 20–65)	Reduced skeletal muscle mass in 17.1%, linked to longer T1D duration and higher retinopathy prevalence (58.8% in those with low skeletal mass vs 15.9% in those with normal skeletal mass) Retinopathy, male sex and BMI were independent risk factors
Pollakova (2023), cohort study [75]	To evaluate the prevalence of sarcopenia in younger adults with long-standing T1D	T1D	39	Mean: 49.3	Sarcopenia was present in 7.7% of participants with T1D (12.5% in women, 4.35% in men), while 23.1% had low appendicular lean mass index Low appendicular lean mass was associated with longer disease duration, higher fat mass and poorer muscle strength
Hiromine (2022), cross‐sectional study [73]	To investigate differences in the prevalence rates of sarcopenia and its components between individuals with T1D and T2D	T1, TD2	812 (T1D *n*=57, T2D *n*=755)	Mean: ≥65 (mean ± SD: T1D 62.7 ± 12.3, T2D 69.9 ± 9.0)	Sarcopenia: higher in T1D vs T2D (20.0% vs 8.1%) Low handgrip strength: higher in T1D (50.0% vs 28.7%) T1D identified as a significant risk factor for low strength Compared with healthy control participants, people with T1D had reduced muscle thickness and strength Poor glucose management (not insulin use) linked to sarcopenia
Tan (2022), cross-sectional study [72]	To compare muscle strength and architecture in individuals with and without TD1	T1D	32	Mean ± SD: 31.3 ± 8.7	Significantly lower quadriceps muscle thickness in participants with T1D, independent of HbA_1c_ or diabetes duration Individuals using insulin pumps showed more favourable muscle characteristics than those on subcutaneous insulin

T1D, type 1 diabetes; T2D, type 2 diabetes

Early muscle decline is also evident in middle-aged adults with long-standing type 1 diabetes and is related to higher fat mass and microvascular complications [74, 75].

## The role of cognitive impairment and multimorbidity in frailty

As life expectancy of people with type 1 diabetes improves, the prevalence of age-related conditions, including cognitive impairment, is expected to increase. Studies have shown that people with type 1 diabetes may experience significant cognitive deficits, particularly in processing speed, attention, executive function and cognitive flexibility while memory and learning are usually preserved [76]. These deficits may appear as early as midlife, especially in those with childhood-onset diabetes, and worsen with the presence of microvascular complications [76]. Moreover, older individuals with long-standing type 1 diabetes or history of severe hypoglycaemia are at increased risk of developing cognitive impairment and particularly vascular dementia. A recent systematic review and meta-analysis including six cohort studies found that people with type 1 diabetes have a 50% higher risk of developing any form of dementia compared with control individuals (pooled HR 1.50 [95% CI 1.25, 1.80], *p*<0.001) [77].

There appears to be an association between severe hypoglycaemia and cognitive impairment in older adults with type 1 diabetes and this has been corroborated by data from the Study of Longevity in Diabetes (SOLID) [78]. In SOLID, including 718 individuals (mean age 67.2 years), severe hypoglycaemia within the past year was associated with deficits in language, executive function and episodic memory and tripled the odds of impaired global cognition (OR 3.22) [78].

Cognitive impairment can have various consequences in individuals with type 1 diabetes, including inability to self-care [79] and increased risk of severe hypoglycaemia, which may lead to further functional decline and loss of independence [79]. Dementia and type 1 diabetes negatively influence each other, as cognitive decline reduces self-management capacity while hypo- and hyperglycaemia can accelerate cognitive deterioration, highlighting the need to adapt treatment strategies. Given that cognitive impairment is both a manifestation and a driver of frailty, its early identification and assessment are key processes in the care of older adults with type 1 diabetes.

## Frailty assessment

Early recognition of frailty in people with type 1 diabetes is an important first step to support effective management. A number of frailty assessment tools have been introduced into practice [80] and include those based on patient-reported information or on the clinician’s assessment, and those relying on electronic health records [80]. Table 2 lists some of the established tools. The clinical frailty scale (CFS) and the electronic frailty index (eFI) are the most-used frailty assessment tools in the community setting in the UK.

**Table 2:** Commonly used frailty tools in clinical settings

Frailty assessment tool	Type of assessment	Components	Frailty classification	Predictive outcomes
Fried frailty phenotype	Clinician’s subjective assessment	Five criteria: weight loss; low physical activity; exhaustion; slowness; weakness	Frailty: ≥3 criteria Pre-frailty: 1–2 criteria Robust: 0 criteria	Disability, falls, hospitalisation, mortality [6]
CFS	Clinician’s subjective assessment	Pictographic nine-point scale: 1=very fit; 9=terminally ill	Frailty: score ≥5	Mortality (30 days, 1 year), hospitalisation, ICU admission [136, 137]
FRAIL scale	Patient self-report	Five criteria: fatigue; resistance; ambulation; illness; loss of weight	Frailty: ≥3 criteria Pre-frailty: 1–2 criteria Robust: 0 criteria	Surgical risk, short term, morbidity [138]
Frailty index	Medical record review	Thirty or more health deficits: scores range from 0 (no deficits) to 1 (all deficits)	Continuous score; suggested cut-off score for frailty ≥0.25	Mortality, disability, institutionalisation, hospitalisation [139, 140]
eFI	Medical record review	As for the frailty index, with variables derived from routine electronic health records in primary care; also considered to be a case-finding instrument	Severe frailty: score >0.36 Moderate frailty: score >0.24–0.36 Mild frailty: score >0.12–0.24 Fit: score ≤0.12	Hospitalisation, care home admission, mortality [82, 141]

ICU, intensive care unit

The CFS is a nine-point visual scale classifying individuals from very fit to terminally ill and predicts adverse outcomes such as institutionalisation and mortality [81]. The eFI is derived from UK primary care data routinely collected in clinical practice and is based on the cumulative deficit model. It classifies individuals into fit and mildly, moderately and severely frail and predicts transition to care homes, hospital admission and death [82].

In addition, a number of simple screening and performance-based assessments such as PRISMA-7, gait speed, the Timed Up-and-Go test and grip strength are used in clinical and research settings to identify individuals at risk of frailty or to evaluate specific functional domains and predict a broad range of unfavourable outcome [83–87].

## Why assess frailty?

Frailty assessment should be considered as an essential part of comprehensive, person-centred diabetes care [3]. A holistic approach incorporating multiple domains represents a necessary evolution from the traditional model of care focused on glycaemic management and the prevention and treatment of complications, and is essential to identify older adults with type 1 diabetes at increased risk of adverse outcomes.

Frailty is strongly associated with a range of clinician-reported outcomes including disability, hospital admissions, mortality and diabetes-related complications such as hypoglycaemia and microvascular complications [14]. Frailty is also linked to a substantially increased risk of falls and fractures, which are key drivers of morbidity, loss of independence and long-term care needs in older adults. In our previous work we demonstrated that frailty, as estimated using the frailty index, was highly prevalent in a cohort of individuals with type 1 diabetes and type 2 diabetes and diabetic foot ulcers and predicted non-healing of wounds and re-hospitalisation [88].

Frailty is also associated with patient-reported outcomes such as fatigue, reduced quality of life and loss of independence, as well as care process-related outcomes, including longer hospital stays, delayed discharges and increased need for community support [89].

**Fig. 2:**
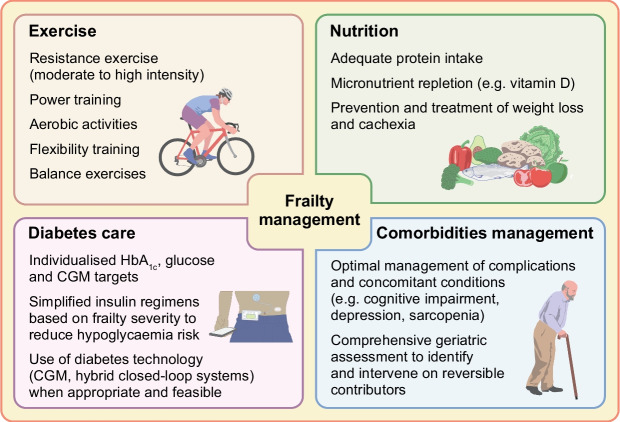
Frailty management in type 1 diabetes: core domains. This figure is available as part of a downloadable slideset

Identification of frailty and a holistic evaluation of the older person with type 1 diabetes can guide tailored treatment strategies that target modifiable factors to prevent, delay or even reverse frailty progression (Fig. 2).

## Interventions for frailty

Frailty is a dynamic condition that can stabilise, progress or improve [90]. Established frailty interventions include exercise, nutrition, and multicomponent or individually targeted strategies. Exercise and nutritional supplementation either alone or in combination can improve the frailty phenotype in community-dwelling older adults [11].

Exercise is a cornerstone in the prevention and improvement of frailty and is known to improve balance, muscle strength and functional ability [91]. Current evidence supports the use of exercise programmes including aerobic, resistance, power, flexibility and balance training two or three times a week for 45–60 min for at least 3 months [92]. Resistance training is a key component and the most effective standalone approach to combat frailty. It involves exercises using external resistance (e.g. weights, machines, bands or water) [92]. Mind–body practices such as yoga and tai chi have been shown to improve mobility, chair-rise ability, balance and low extremity function but not muscle mass or grip strength [93, 94]. Tai chi also reduces falls and fear of falling, and enhances mood, cognition and quality of life [94].

Medication optimisation, through review and dose reduction or withdrawal of agents that are potentially harmful or have minimal benefits, may help lower mortality risk and slow down functional decline [95, 96]. In the care home setting, medication optimisation has been associated with a reduced risk of falls and fewer hospital admissions [97].

Nutritional interventions, ensuring adequate protein intake and addressing unintentional weight loss, are crucial to maintain muscle mass and function. When combined with protein supplementation, resistance training promotes muscle hypertrophy and enhances both muscle mass and strength. Because of age-related anabolic resistance, in older adults, proteins should represent up to 20–30% of total energy intake or 1.0–1.2 g/kg per day [98]. For frail individuals, current guidelines recommend a daily protein intake of 1.2–1.5 g/kg [92].

Moreover, identifying and addressing modifiable risk factors such as vitamin deficiencies, anaemia and suboptimal management of long terms conditions can contribute to improving frailty status (Fig. 2). Anaemia due to vitamin B_12_ and folate deficiencies or related to chronic kidney disease, which is common in diabetes, can worsen fatigue and physical and cognitive decline. In contrast, supplementation with vitamin D, *n*-3 fatty acids, sex hormones or growth hormone has shown minimal impact on frailty [11].

Given the multifactorial nature of frailty, strategies combining multiple interventions are more effective, particularly in pre-frail individuals. The superiority of multimodal approaches is supported by systematic reviews and meta-analyses reporting greater improvements in muscle mass and strength and physical performance [99].

The MIDFRAIL trial evaluated the impact of a multimodal intervention including progressive resistance training, structured diabetes and nutrition education and optimisation of glucose and cardiovascular targets in frail and pre-frail older adults with type 2 diabetes [100]. Participants assigned to this programme for 16 weeks, experienced significant improvements in physical performance and frailty status, as measured by the Short Physical Performance Battery, when compared with participants in the usual-care group. Follow-up data showed that these benefits were sustained for up to 24 months after the intervention [101]. To date there are no randomised trials assessing frailty interventions in older adults with type 1 diabetes but it is highly likely that multimodal interventions targeting exercise, nutrition and treatment optimisation and supplemented by diabetes technology may help preserve function and delay frailty progression in this population.

## Novel potential therapeutic approaches to manage frailty

Some oral glucose-lowering agents traditionally used in type 2 diabetes may potentially influence frailty through slowing muscle loss and reducing the risk of falls [102, 103]. Metformin is already used in type 1 diabetes, particularly in individuals with overweight and features of insulin resistance. Its pleiotropic effects, including improvements in insulin sensitivity, reduction of oxidative stress and inflammation and modulation of cellular pathways involved in ageing, make this drug an attractive option to prevent the onset and progression of frailty [104].

Similarly, sodium–glucose cotransporter 2 inhibitors (SGLT2i) have been shown to improve cardiovascular and renal outcomes in individuals with and without diabetes and have captured attention for their anti-ageing properties [105]. In a previous review, we described their defence against frailty and their pro-longevity effects mediated by anti-ageing hormones and transcription factors and genes, including attenuation of oxidative stress and inflammation and improvement of metabolism [105]. Although they are not currently approved or recommended for use in people with type 1 diabetes, ongoing trials are exploring their potential cardiovascular and renal benefits in this group [106]. Continuous ketone monitoring, currently under investigation in clinical trials, may in future enable the safe use of SGLT2i in people with type 1 diabetes (ACTRN 12624000992505). Alongside these benefits, there is conflicting data on the impact, if any, of SGLT2i on skeletal muscle health and the mechanisms involved remain unclear. Many of the studies are short term and with heterogenous results. Meta-analyses in type 2 diabetes have consistently shown small reductions in lean or skeletal muscle mass or muscle strength associated with SGLT2i-induced weight loss [107, 108]. In contrast, real-world studies have shown favourable changes in body composition when muscle mass is considered relative to fat mass, as well as preservation of muscle strength and function [109, 110]. The mechanisms by which SGLT2i affect muscle mass are diverse and may reflect the heterogeneity of data reported in the literature [111]. On the one hand, SGLT2i trigger glycosuria, thereby promoting mild catabolism, reduced muscle glucose uptake and increased proteolysis [111]. On the other hand, SGLT2i enhance ketogenesis, improve mitochondrial efficiency and decrease inflammation and oxidative stress, mechanisms that may preserve muscle function [111]. Overall, the available data are inconclusive and highlight the need for studies looking at functional outcomes, particularly in frail older people with type 1 diabetes.

Ageing with type 1 diabetes may be accompanied by increasing adiposity and insulin resistance, potentially supporting the use of glucagon-like peptide-1 receptor agonists (GLP-1RAs) in selected individuals. Beyond their established effects on weight and glycaemic measures [112, 113], preclinical studies show that GLP-1RAs reduce systemic inflammation and oxidative stress, support mitochondrial function and exert neuroprotective actions, with potential benefits in age-related conditions such as Parkinson’s disease and Alzheimer’s disease [114, 115]. It should be acknowledged that GLP-1RA-induced weight loss may include a degree of lean mass reduction, raising concerns about sarcopenia. Reassuringly, emerging mechanistic, imaging and preclinical data suggest that GLP-1RAs may improve muscle composition by reducing intramuscular fat and may exert anti-inflammatory, mitochondrial and neuroprotective effects that could help preserve muscle quality and function [116]. These benefits appear to occur through the modulation of key signalling pathways, including PI3K–Akt–mTOR and AMPK–PGC-1α, as well as through the suppression of proteolytic activity and the promotion of myogenic differentiation [116]. However, while GLP-1RAs may offer a means of mitigating the burden of double diabetes and supporting metabolic and pleiotropic benefits, dedicated randomised trials in older or frail individuals with type 1 diabetes are still lacking. In clinical practice, it is therefore advisable to select GLP-1RA (or dual glucose-dependent insulinotropic polypeptide [GIP]/GLP-1RA) regimens with modest potency, to use slower titration schedules, or to combine treatment with resistance exercise and dietary strategies that ensure adequate protein intake. In older adults, particularly those with sarcopenic obesity, the initiation of GLP-1RA therapy should follow a careful clinical assessment, include screening for sarcopenia and be accompanied by ongoing monitoring of physical function. In addition to these adjuvant treatments for type 1 diabetes, senolytics and geroprotectors are emerging as potential therapies for frailty. Senolytics, such as dasatinib and quercetin, selectively clear senescent cells, thereby reducing inflammation and metabolic dysfunction in animal models [117]. Agents such as rapamycin and resveratrol have shown the potential to mitigate frailty and delay the onset of age-related pathologies and may hold relevance for individuals with multimorbidity, including those with diabetes, by acting on multiple pathophysiological pathways [118].

## Glycaemic targets in older adults with type 1 diabetes according to frailty status

**Fig. 3:**
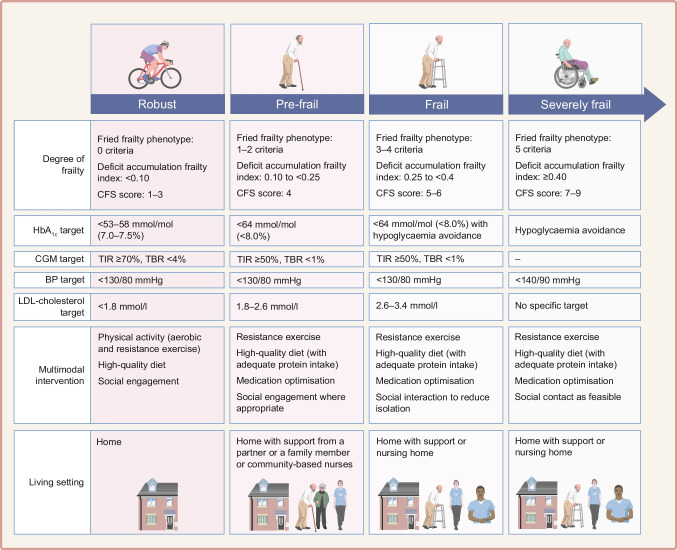
Holistic approach to older people with type 1 diabetes according to frailty status. A frailty-based framework for older adults with type 1 diabetes is presented, including glycaemic, BP and lipid targets across frailty categories. Glycaemic targets incorporate both HbA_1c_ and CGM metrics. The framework also integrates care setting and multimodal interventions to support a person-centred approach to care. TBR, time below range (<3.9 mmol/l); TIR, time in range (3.9–10.0 mmol/l). This figure is available as part of a downloadable slideset

Clinical guidelines recommend that HbA_1c_ targets in older adults with type 1 diabetes should be tailored according to health status, number of comorbidities, severity of frailty and life expectancy [119, 120]. In robust individuals, HbA_1c_ targets are <53–58 mmol/mol (<7.0–7.5%); in pre-frail and frail individuals, HbA_1c_ targets are set at <64 mmol/mol (<8%) with a particular emphasis on hypoglycaemia avoidance in the frail group [119, 120] (Fig. 3). In contrast, in individuals with severe frailty and limited life expectancy, the primary aim is avoiding hypoglycaemia rather than attaining specific HbA_1c_ targets [119, 120]. However, a higher HbA_1c_ level does not preclude the risk of hypoglycaemia [121]. HbA_1c_ reflects average glucose levels and may not capture glucose variability or episodes of asymptomatic hypoglycaemia [121].

In frail older adults, prolonged hyperglycaemia can be detrimental and can potentially contribute to fatigue, functional and cognitive decline, acute kidney injury and recurrent urinary infections. These complications can potentially accelerate frailty. Continuous glucose monitoring (CGM) can be particularly useful in this population as it enables identification of hypo- and hyperglycaemia and tailored and safer glycaemic management.

Over the past few years, there has been an increasing use of CGM. Several prospective and retrospective studies have clearly shown that use of these technologies in people with diabetes is associated with improved glucose management, reduced glucose variability, reduction in episodes of hypoglycaemia and fewer emergency department visits for hypoglycaemia and hyperglycaemia [122]. In 2019, an international consensus group led by Battelino defined metrics for CGM data interpretation [123]. The panel of experts identified a group at ‘high risk’, including older adults with type 1 diabetes and type 2 diabetes, for which they recommended a less-stringent time in range (3.9–10.0 mmol/l glucose) target of >50% (vs >70% in younger adults) and a time below range (<3.9 mmol/l) target of <1% (vs <4% in younger adults), irrespective of type of diabetes, functional status and frailty. Toschi et al challenged the consensus CGM targets for older adults, highlighting that a time in range goal of >50% (8.3%) is inappropriately lax for non-frail older adults using modern technologies [124]. They proposed stratifying targets by health status (healthy, intermediate, poor) in line with the ADA guidelines [124] and stress that minimisation of hypoglycaemia risk should not be achieved at the expense of time in range as this would lead to worsened hyperglycaemia and glucose variability [124].

A novel concept is the ‘hypoglycaemia buffer zone’ (3.9–5.0 mmol/l glucose in healthy individuals, 3.9–5.6 mmol/l in intermediate/poor health) to allow early detection and timely treatment of hypoglycaemia and prevention of rebound hyperglycaemia [124]. Toschi et al also recommend high upper limits for time in range (≤11.1 mmol/l glucose in the intermediate group, ≤13.9 mmol/l in the poor health group) and adjustments in time above range goals [124]. Finally, they suggest that the Glyaemia Risk Index, which integrates both hypoglycaemia and hyperglycaemia, may better capture the glycaemic burden than time in range alone, especially in frail older adults [124].

In addition to individualised glucose targets, lipid and BP goals should also be tailored according to cardiovascular risk, frailty status and life expectancy (Fig. 3).

## Frailty and diabetes technology

Hypoglycaemia is an independent risk factor for the development or progression of frailty in older adults with diabetes [125]. Older adults with long-standing type 1 diabetes are particularly susceptible to episodes of hypoglycaemia, due to impaired physiological counterregulatory responses [126]. Preventing or minimising hypoglycaemic events is therefore a critical strategy to prevent frailty or influence its trajectory.

Modern diabetes technology such as CGM, insulin pumps and hybrid closed-loop systems can offer substantial benefits for selected older adults with type 1 diabetes, including improved time in range, reduced hypoglycaemia and potentially fewer diabetes-related hospitalisations [122].

Although most evidence comes from functionally independent individuals, early data suggest that these technologies may also be feasible in older adults with mild cognitive or physical impairments [122]. However, implementation in these groups requires a comprehensive assessment to ensure safety, usability and alignment with the individual’s goals of care. Recent evidence supports integrating geriatric principles into diabetes technology use [127, 128]. The TANGO trial demonstrated that combining CGM with simplified treatment regimens and personalised glycaemic targets significantly reduced time spent in hypoglycaemia without worsening overall glycaemic management in adults with type 1 diabetes and recurrent hypoglycaemia [127]. This geriatrics-based model provides a useful framework for the use of CGM in older adults at risk of frailty, where minimising therapy complexity and hypoglycaemia burden is key.

At present, the uptake of diabetes technology by frail individuals remains limited due to barriers such as reduced dexterity, cognitive decline, digital illiteracy and lack of caregiver or support from healthcare professionals with the requisite expertise [122]. Overcoming these challenges requires tailored education, caregiver involvement, simplified device interfaces and collaboration between diabetes and geriatrics teams.

## Living settings of a person with type 1 diabetes and frailty

The living setting of individuals with type 1 diabetes and frailty is often determined by the severity of frailty and the level of assistance required [3]. Some individuals with type 1 diabetes and frailty can continue to live at home with the support of family members or a carer who help manage their diabetes and assist with daily tasks. Carers and family members also play a crucial role during in-person or remote clinic consultations, providing important information and assisting in decision-making.

For frail individuals who are unable to care for themselves and still live at home, insulin administration and glucose testing are often carried out by a family member or district nurses. District nurses (community-based registered nurses providing home visits for clinical care in the UK) support housebound frail people with the administration of medications, including insulin. The involvement of district nurses can, however, come with certain limitations such as inappropriate simplification of insulin regimens, resulting in suboptimal glycaemic management, increased risk of hypoglycaemia and insulin being administered far from mealtimes, all of which can complicate the challenges of managing type 1 diabetes in frail individuals. Ensuring that insulin regimens are both feasible and effective for this population remains an area for improvement.

For frail individuals without family support or carers, transition to long-term facilities may be necessary. Some of our previous work has unveiled that care home staff are often inadequately trained in diabetes management, with consequences for residents with type 1 diabetes [129]. Specialised diabetes training for care home staff has long been advocated but progress has been very limited and large gaps in care quality persist [130, 131].

## Frailty in young individuals with type 1 diabetes

Although frailty is conceptualised as a geriatric syndrome it is not exclusive to older adults. In clinical practice, we increasingly encounter younger adults with type 1 diabetes who exhibit clinical features suggestive of frailty. These individuals have a long-standing suboptimally managed diabetes, have disabling advanced microvascular complications and are often on dialysis. Early-onset frailty, which differs from age-related frailty, has equally important implications in terms of functional status, quality of life and health outcomes.

At present there are no available data on the prevalence, incidence and trajectory of frailty in younger populations with type 1 diabetes. Furthermore, the frailty assessment tools used in both research and clinical settings have been developed and validated in adults over the age of 65 years and may not take into consideration early-life risk factors for frailty. Consequently, at present, our ability to identify, stratify or manage frailty in younger individuals is very limited. This represents a critical clinical and research gap, as younger adults with type 1 diabetes often have decades of the condition burden ahead of them.

## Integrated care approaches for frail older adults with type 1 diabetes

Preventing, delaying or potentially reversing frailty in older adults with type 1 diabetes requires an integrated, person-centred, multidisciplinary approach. Routine frailty screening should be part of diabetes care using validated, time-efficient and user-friendly tools. Early identification of pre-frailty or frailty offers a key opportunity to intervene before significant functional decline occurs. To adopt this strategy, community-based diabetes teams would benefit from training in the recognition of frailty.

Leveraging health data, including electronic health records, CGM, wearable technologies and remote monitoring devices can facilitate the early identification of glucose variability or hypoglycaemia frequency and changes in physical or cognitive function [132–134]. Artificial intelligence-enabled risk stratification could enhance this process [135].

Although diabetes clinicians may adopt basic tools to assess cognition, nutrition and physical function, the most effective and sustainable model of care for older adults with type 1 diabetes is multidisciplinary. The complexity of frailty warrants coordinated care delivered by geriatricians, physiotherapists, dietitians, psychologists, diabetes specialist nurses and social care professionals. Establishing clear referral pathways to geriatric services is a priority to guarantee continuity across primary, specialist and social care.

## Future research directions

As the population of adults with type 1 diabetes continues to grow older, more research in this area is warranted. Limitations in our understanding of the interactions between frailty, ageing and type 1 diabetes, together with the failure of studies to distinguish between type 1 and type 2 diabetes, further contribute to knowledge gaps in this field. Future research should include longitudinal studies exploring the natural progression and risk factors of frailty in type 1 diabetes, as well as well-designed clinical trials evaluating the effectiveness of multimodal interventions in preventing progression of frailty. Studies should recruit individuals with complex profiles including risk of hypoglycaemia, impaired hypo-awareness, polypharmacy and geriatric syndromes.

Additionally, there is a pressing need to investigate early-onset frailty in younger individuals with type 1 diabetes and to develop or validate frailty assessment tools that can be used in this age group.

Finally, given the advancements in diabetes technology, clinical trials will need to evaluate the effect of CGM devices and automated insulin delivery systems on frailty and outcomes of interest to older adults.

## Conclusions

Type 1 diabetes can contribute to frailty through micro- and macrovascular complications leading to sarcopenia and age-related comorbidities such as cognitive impairment. Despite the progressive growth of the type 1 diabetes population at risk of frailty, this remains an under-researched area with significant knowledge gaps. Frailty in the context of type 1 diabetes presents unique challenges and requires novel multidisciplinary models of care that integrate cognitive, physical and social domains and are aimed at improving functional outcomes.

Future clinical strategies should focus on the early identification of frailty in people with type 1 diabetes, promoting individualised treatment goals and delivering multimodal interventions to improve outcomes in this population.

## Supplementary Information

Below is the link to the electronic supplementary material.

Slideset of figures (PPTX 695 KB)

## Abbreviations

CFS: Clinical frailty scale
CGM: Continuous glucose monitoring
eFI: Electronic frailty index
GLP-1RA: Glucagon-like peptide-1 receptor agonist
SGLT2i: Sodium–glucose cotransporter 2 inhibitor
SOLID: Study of Longevity in Diabetes
