# Supplementary material for: Type 1 diabetes, ageing and frailty: an underexplored intersection
Source: Diabetologia. 2026 Feb 17;69(5):1133–49. doi: 10.1007/s00125-026-06681-x (PMC13005784; doi:10.1007/s00125-026-06681-x)
Supplement: Supplementary file 1 — Slideset of figures (PPTX 695 KB) [file 125_2026_6681_MOESM1_ESM.pptx]

## Slide 1
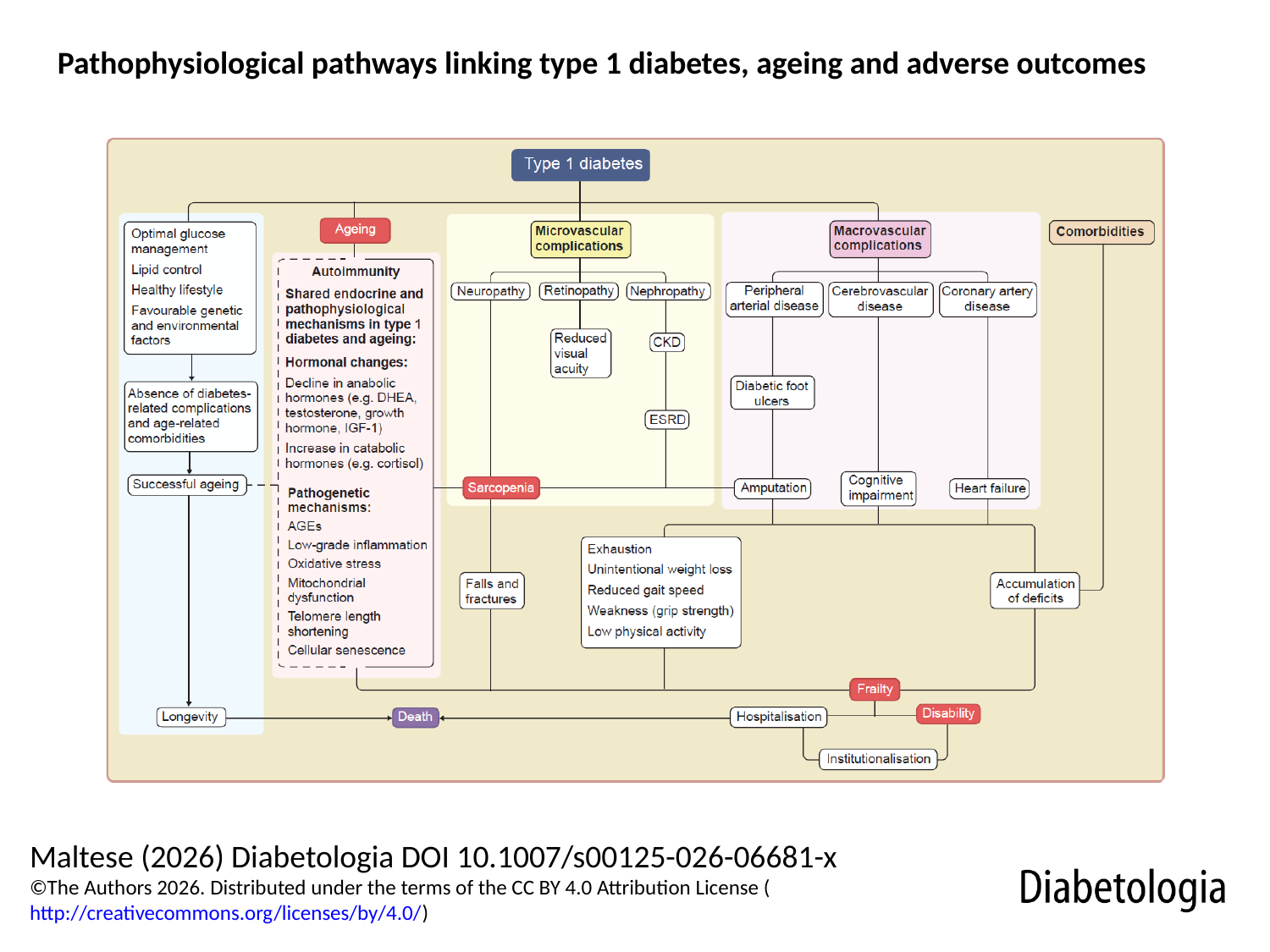

Pathophysiological pathways linking type 1 diabetes, ageing and adverse outcomes
Maltese (2026) Diabetologia DOI 10.1007/s00125-026-06681-x
©The Authors 2026. Distributed under the terms of the CC BY 4.0 Attribution License (http://creativecommons.org/licenses/by/4.0/)

## Slide 2
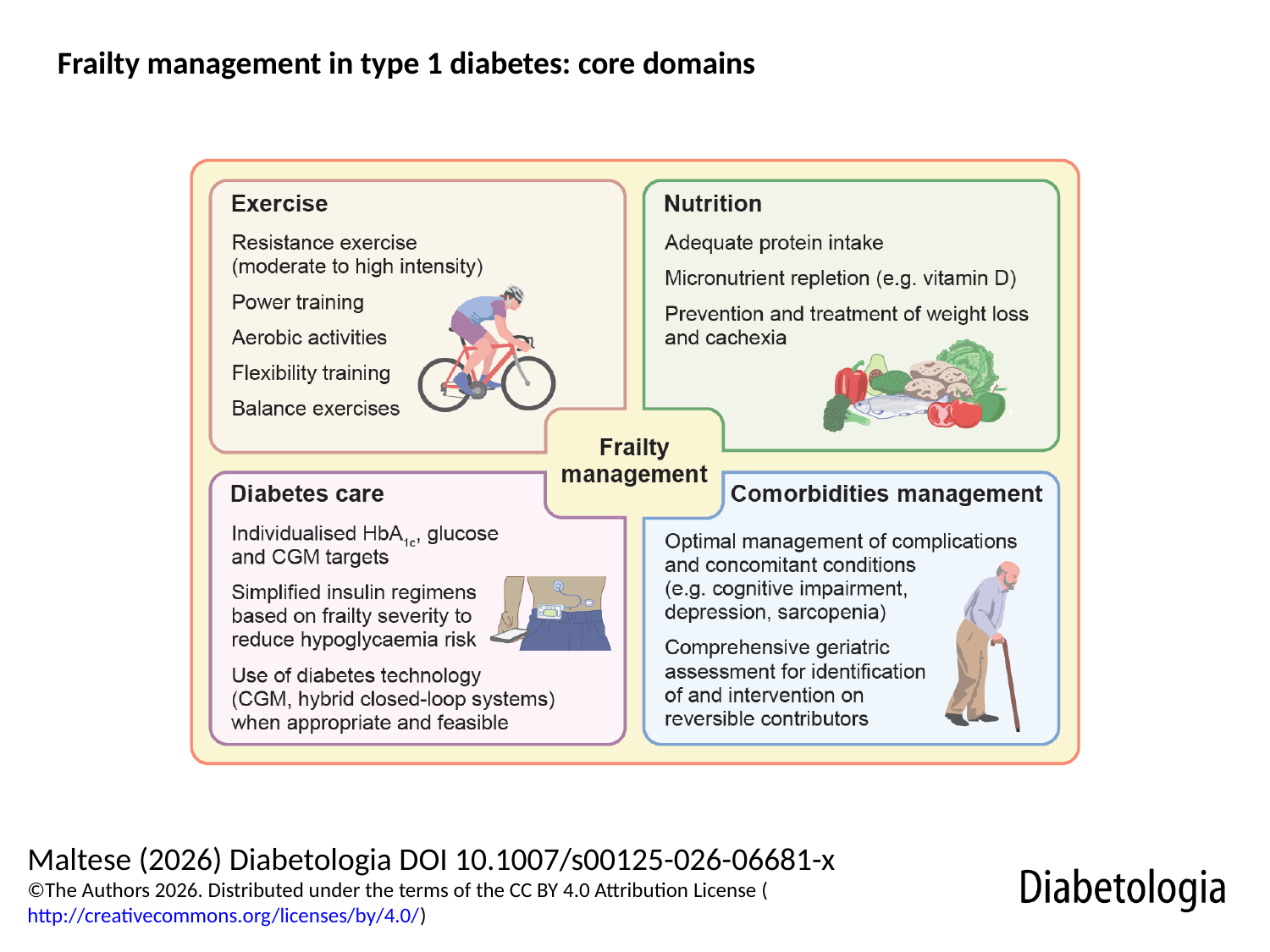

Frailty management in type 1 diabetes: core domains
Maltese (2026) Diabetologia DOI 10.1007/s00125-026-06681-x
©The Authors 2026. Distributed under the terms of the CC BY 4.0 Attribution License (http://creativecommons.org/licenses/by/4.0/)

## Slide 3
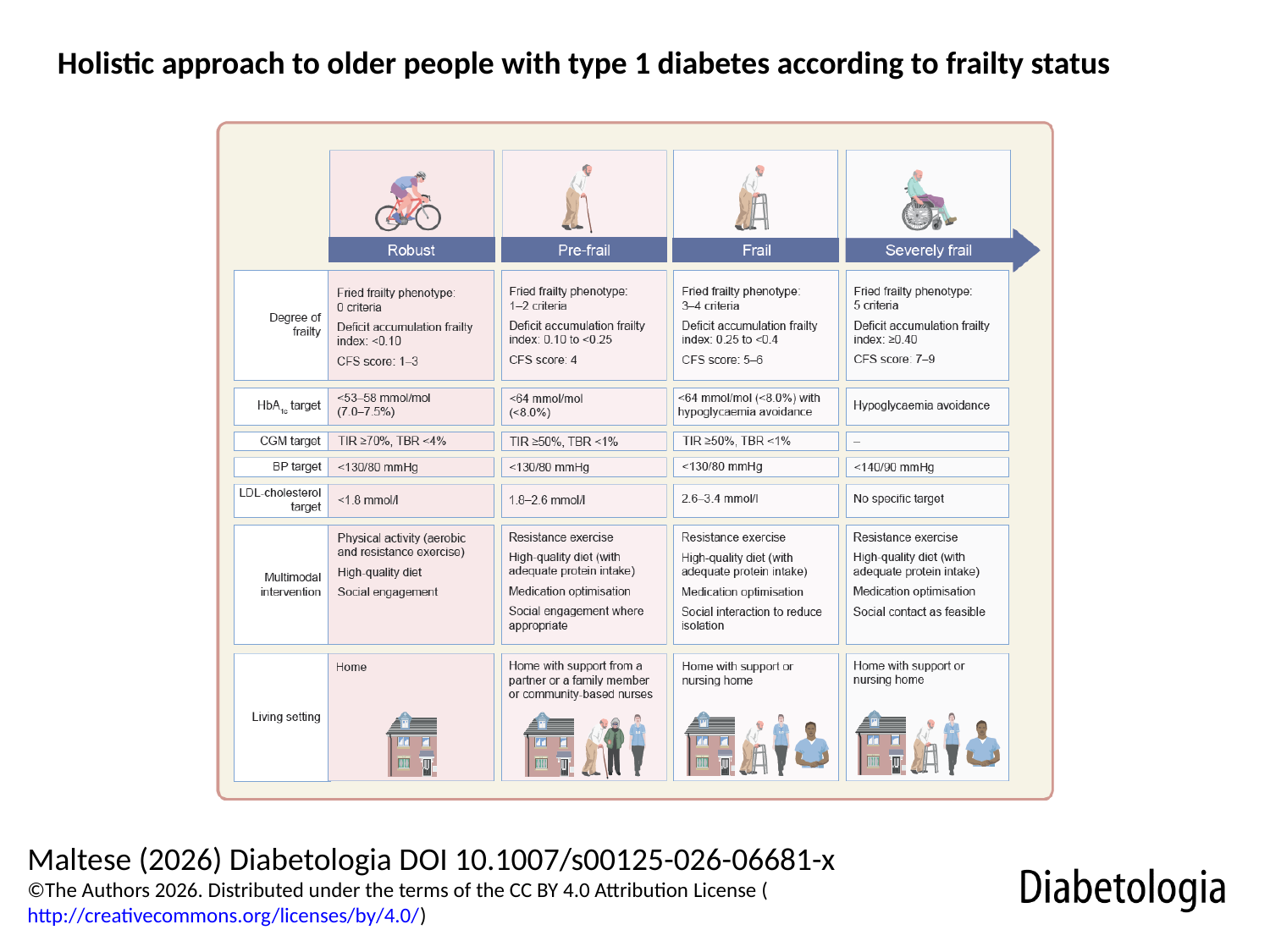

Holistic approach to older people with type 1 diabetes according to frailty status
Maltese (2026) Diabetologia DOI 10.1007/s00125-026-06681-x
©The Authors 2026. Distributed under the terms of the CC BY 4.0 Attribution License (http://creativecommons.org/licenses/by/4.0/)
